# Research and Application of Fine-Grained Image Classification Based on Small Collar Dataset

**DOI:** 10.3389/fncom.2021.766284

**Published:** 2022-04-11

**Authors:** Huang Chengcheng, Yuan Jian, Qin Xiao

**Affiliations:** ^1^Guangxi Key Lab of Human-Machine Interaction and Intelligent Decision, Nanning Normal University, Nanning, China; ^2^Guangxi International Businiess Vocational College, Nanning, China; ^3^Guangxi University for Nationalities, Nanning, China

**Keywords:** convolutional neural network, collar classification, clothing classification, attention mechanism, loss function

## Abstract

With the rapid development of apparel e-commerce, the variety of apparel is increasing, and it becomes more and more important to classify the apparel according to its collar design. Traditional image processing methods have been difficult to cope with the increasingly complex image backgrounds. To solve this problem, an EMRes-50 classification algorithm is proposed to solve the problem of garment collar image classification, which is designed based on the ECA-ResNet50 model combined with the MC-Loss loss function method. Applying the improved algorithm to the Coller-6 dataset, and the classification accuracy obtained was 73.6%. To further verify the effectiveness of the algorithm, it was applied to the DeepFashion-6 dataset, and the classification accuracy obtained was 86.09%. The experimental results show that the improved model has higher accuracy than the existing CNN model, and the model has better feature extraction ability, which is helpful to solve the problem of the difficulty of fine-grained collar classification and promote the further development of clothing product image classification.

## Introduction

In recent years, due to the emergence of convolutional neural networks, deep learning has been applied more and more widely, including image recognition and natural language processing (Wu et al., 2018; Yuan et al., 2019; Qin et al., 2020; Wu Y. et al., 2020). Wu E. Q. et al. (2019) proposed a Fuzzy Gaussian Support Vector Machine (FGSVM) as a top-level classification tool for deep learning models in order to more accurately classify the pilot's attention state images and analyze the abnormal conditions of the pilot's flight state. Eliminate some Gaussian noise output by the Deep HCAE Network (DHCAEN), which effectively improves the accuracy of image classification. Wu E. Q. et al. (2020) proposed a gamma deep belief network to extract multi-layer depth representation of high-dimensional cognitive data in order to solve the problem of inaccurate identification of pilot fatigue state, and realized automatic reasoning of network structure, with satisfactory results of model accuracy.

In addition, with the advent of the global Internet era, people only need an Internet electronic device to access the Internet and buy products on e-commerce platforms. Therefore, e-commerce is developing rapidly, and recommendation technologies and applications of e-commerce have also attracted the attention of many researchers. For the QoS, Wu D. et al. (2019) propose a posterior-neighborhood-regularized LF (PLF) model for achieving highly accurate Quality-of-Service (QoS) prediction for web services. Wu D. et al. (2020) proposed a data-characteristic-aware latent factor (DCALF) model to implement highly accurate QoS predictions. For the recommender systems, Wu et al. (2021b) proposed an L1-and-L_2_-norm-oriented LF (L3F) model, it has good potential for addressing High-Dimensional and Sparse (HiDS) data from real applications. Wu et al. (2021a) proposed a deep latent factor model (DLFM), it can better describe users' preferences for projects.

Today's popular shopping sites support keyword searches for the style of clothing you want to buy, including keyword searches for clothing collar types. However, product information on websites is often described through a combination of direct image descriptions and key text markups. Text tagging requires a lot of manpower to mark accurately. If images can be directly described, a lot of time and labor costs can be reduced. In the traditional classification and recognition methods of clothing attributes, the amount of feature extraction is huge, and the artificial visual features cannot meet the requirements of real classification, and the efficiency is not high. Therefore, the convolutional neural network in deep learning can be used to efficiently recognize clothing images.

Currently, most researchers focus on apparel category classification or multi-attribute image classification based on apparel. Inoue et al. (2017), in order to solve the multi-label classification problem of fashion images and learn from noisy data unsupervised, provided a new dataset of weakly labeled fashion images of full-body poses Fashion550K with labels containing significant noise and proposed a multi-task label cleaning network to predict the color of clothing and the class of clothing worn by the person in each image. The method generates accurate labels from noisy labels and learns more accurate multi-label classifiers from the generated labels, which effectively solves the multi-label classification problem for fashion images. Liu et al. (2016) collected 800,000 garment images to build a dataset DeepFashion and proposed a deep model of FashionNet based on VGG16, which not only utilizes the attributes and category information of garments but also uses the key point location (landmarks) to assist in extracting features, which can better cope with the deformation of garments It is an effective way to classify clothing styles and attributes. Nawaz et al. (2018) considered the growing market share of online shopping malls and wide popularity of online sales, collected 1,933 images of five different garments from different online stores and retailers' websites to define the traditional garments of Bangladesh and labeled them accordingly, classified the traditional garments using Google Inception based CNN model and used three different optimizers (SGD, Adam, and RmsProp) to test the constructed models. Among these optimizers, RmsProp performs the best.

Most researchers focus on clothing category classification or clothing multi-attribute image classification. There are very few studies on collar image classification and related datasets are not publicly available. Such image classification is more challenging than ordinary image classification because the differences between classes tend to focus on only a small area.

In this paper, we take advantage of the Efficient Channel Attention (ECA)-ResNet50 network model based on the attention mechanism to continuously focus on the most discriminative regions to achieve image classification and combine Mutual-Channel loss (MC-Loss) to make the original collar image focus on more discriminative regions to improve the model classification effect. The main contributions of this paper are as follows:

(1) A clothing collar classification image dataset named Collar-6 was established, which contains 6 categories of the round collar, lapel, stand-up collar, hood, V-neck, and fur lapel, with a total of 18,847 images. The dataset has different degrees of noise interference.
(2) A fine-grained image classification algorithm for a small collar dataset, called ECA MCloss ResNet-50 (EMRes-50), is proposed. experiments are first conducted using the Collar-6 dataset and compared with other popular convolutional neural networks. The experiments do not require any labeled frames and rely only on labels for collar image classification. Second, to verify the effectiveness of the EMRes-50 algorithm, DeepFashion, a public dataset of comparable size to the Collar-6 dataset, is collected for validation. Finally, ablation experiments are performed on EMRes-50. Several experiments have proved that EMRes-50 can effectively solve the problem of collar image classification.

## Related Work

### ECANet

**Figure 1:**
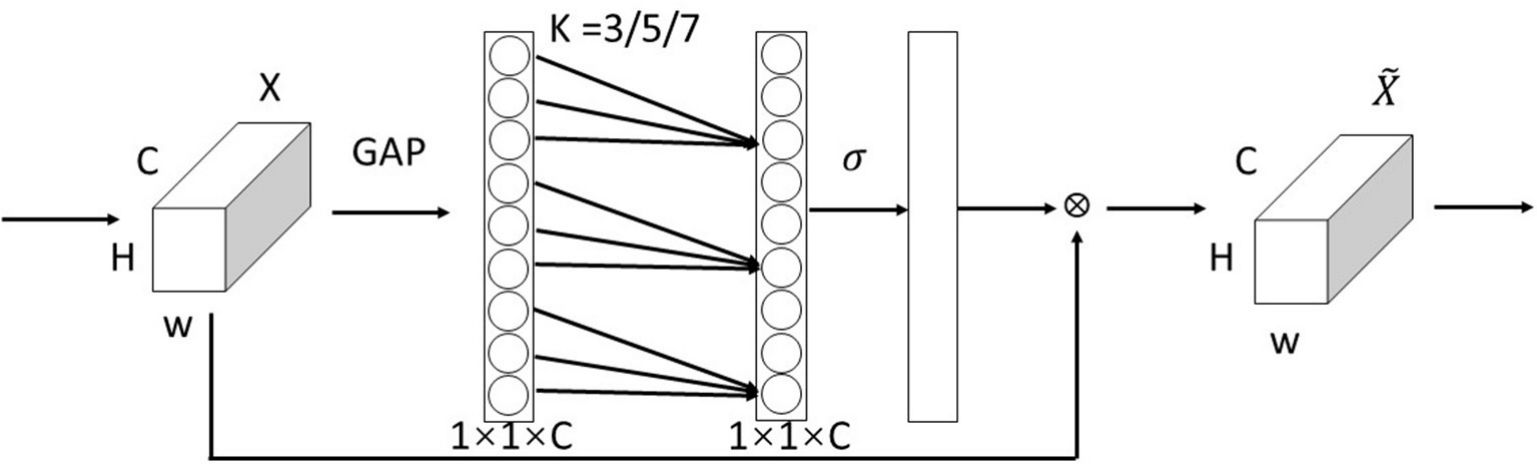
ECA module (Wang Q. et al., 2020).

The visual attention mechanism is unique to visual signal processing in the human brain. After browsing the global image, human vision obtains the visual focus that needs to be focused on, and subsequently devotes more attention resources to this focus region to obtain more detailed information and suppress other useless information, and the Attention Model (AM) (Zhao et al., 2017) of computer vision is generated and has become an important concept in neural networks, which has now been widely used in various types of deep learning tasks such as natural language processing, image recognition and speech recognition (Hu et al., 2018; Woo et al., 2018; Li et al., 2019). The channel attention module assigns different weights to the feature maps, which can be filtered out to help in the classification and attribute prediction of the target. Wang Q. et al. (2020) found by comparing the Squeeze-and-Excitation (SE) module with its three variants Squeeze-and-Excitation Variants 1 (SE-Var1), Squeeze-and-Excitation Variants 2 (SE-Var2), and Squeeze-and-Excitation Variants 3 (SE-Var3) without dimensionality reduction operation that although the design of two fully connected layers in SENet captures the interaction of nonlinear cross-channel information while controlling the complexity of the model, its dimensionality reduction operation is inefficient for capturing the dependencies between all channels. This needs to correspond directly with their weights and avoiding dimensionality reduction is more important than considering the correlation between non-linear channels. Therefore, an efficient channel attention-ECA module for deep convolutional neural networks is proposed, which avoids dimensionality reduction and captures cross-channel information interactions more effectively, allowing the network to selectively enhance informative features, enabling subsequent processing to make full use of these features, and suppressing useless features to ensure computational performance and model complexity. The ECA module is shown in Figure 1.

The ECA module is mainly improved from the SE module (Hu et al., 2018). Using ω_{*k*} to denote the learned channel attention. For the weight *y*_{*i*}, ECANet only considers the information exchange between *y*_{*i*} and *k* neighboring channels, while to further improve the performance, it also allows all channels to share the weight information, as follows:


(1) $$\begin{array}{r} \omega_{i}=\sigma \left(\sum_{j=1}^{k}\omega^{j}y_{i}^{j}\right),y_{i}^{j}\in \Omega_{i}^{k} \end{array}$$

Where, $\Omega_{i}^{k}$ represents the set of *k* adjacent channels of *y*_*i*_. σ is the activation function. ECANet realizes the information exchange between channels through the one-dimensional convolution with the size of the convolution kernel *k*:


(2) $$\begin{array}{r} \omega =\sigma \left({C1D}_{k}\left(y\right)\right) \end{array}$$

Where C1D stands for one-dimensional convolution, and the kernel size *k* represents the coverage of local cross-channel interactions, that is, how many neighbors are involved in the attention prediction of a channel. This method of capturing cross-channel information interactions involves only k parameters, which guarantees performance results and model efficiency. The whole ECA module completes the processing of the attention mechanism in three main steps: First, the global average pooling generates a feature map of 1 × 1 × C size; Second, the adaptive convolution kernel size *k* is computed; Third, *k* is applied in a one-dimensional convolution to obtain the weights of each channel.

**Figure 2:**
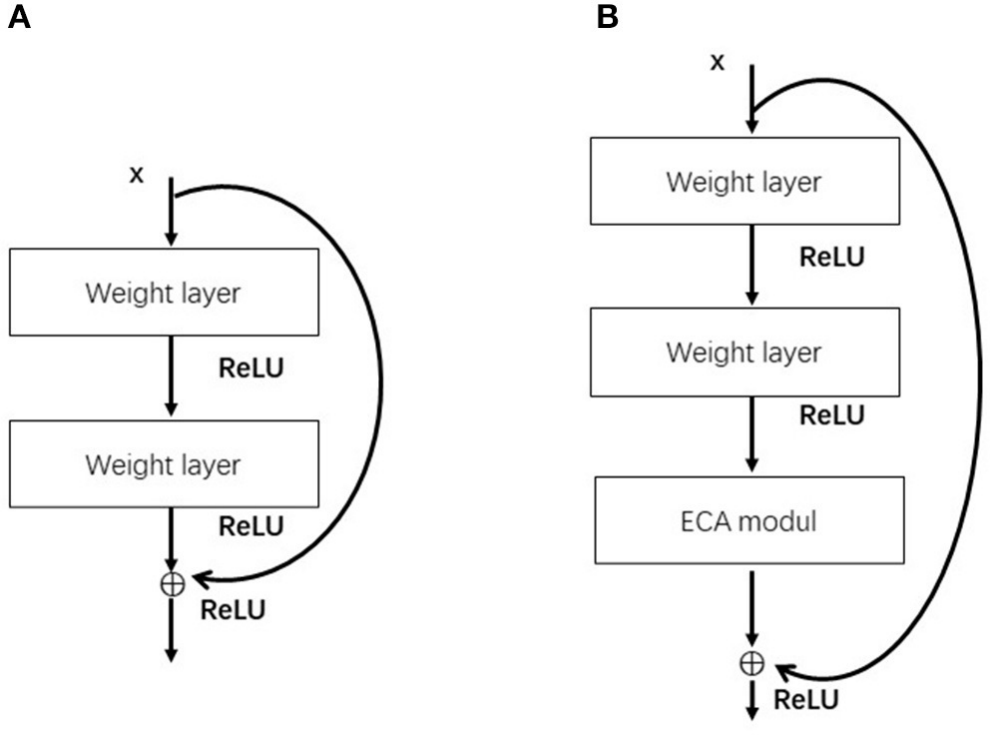
**A** is the residual block, **B** is the residual block introduced into the ECA module.

The ECA module can be flexibly integrated into existing CNN architectures. ECA-ResNet is an improvement for ResNet networks. Figure 2 shows the comparison between the original residual block and the residual block with the introduction of the ECA module. The ECA module is placed after the weight layer in the residual block, and the channel attention is paid to the residual features on the branch before the Addition operation to further increase the feature extraction capability of the network.

### Mutual-Channel Loss

Compared with general image classification tasks, the difference and difficulty of fine-grained image classification tasks are that the granularity of the image category is more refined, and the network model is required to find the distinguishable areas between each sub-category to accurately classify the image category. Chang et al. (2020) proposed Mutual-Channel loss (MC-Loss) to group feature channels, each group uses a fixed number of channels to represent a certain class. The Mutual-Channel loss function can be used in combination with any convolutional neural network model. The MC-Loss function takes the output feature channel of the last convolutional layer as input and aggregates it with the cross-entropy loss function through hyperparameters. The loss function of the final model can be expressed as


(3) $$\begin{array}{r} L=L_{CE}+\mu L_{MC} \end{array}$$

Where, *L*_*CE*_ is the traditional cross-entropy loss function, which makes the network extract the global discriminative region of the image. *L*_*MC*_ is the multi-channel loss function, which makes the network extract the local discriminative region of the image.

**Figure 3:**
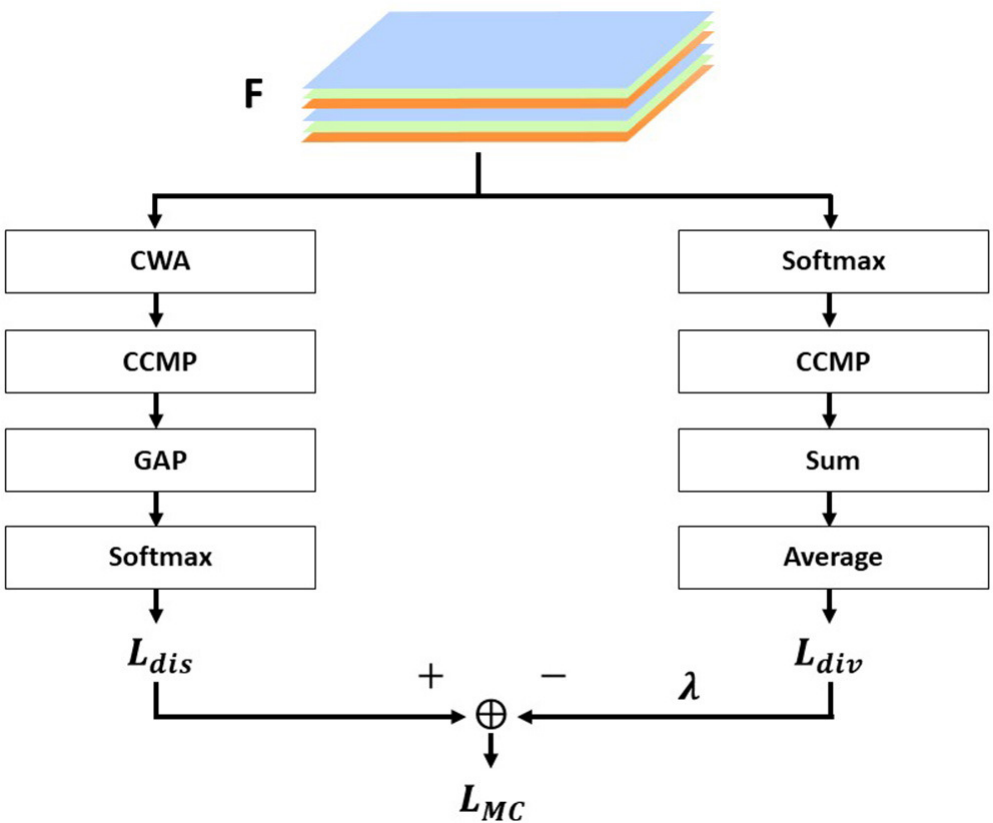
Components of the interpass loss function.

The overall structure of MC-Loss is shown in Figure 3, and the two branches are, respectively, represented as *L*_*dis*_ and *L*_*div*_. *L*_*dis*_ is a discriminality component, and *L*_*div*_ is a diversity component. The feature map extracted from the basic network is *F*∈*R*^*N* × *W* × *H*^.

The *L*_*MC*_ is calculated as follows:


(4) $$\begin{array}{r} L_{MC}\left(F\right)=L_{dis}\left(F\right)-\lambda \times L_{div}\left(F\right) \end{array}$$

In this framework, the discriminative component *L*_*dis*_ is defined as follows:


(5) $$L_{dis}\left(F\right)=L_{CE}(y,\underset{Softmax}{\underset{︸}{\frac{{\left[e^{g(F_{0})},e^{g(F_{1})},\ldots ,e^{g(F_{c-1})}\right]}^{T}}{\sum_{i=0}^{c-1}e^{g(F_{i})}})}}$$

(6) $$g(F_{i})=\underset{GAP}{\underset{︸}{\frac{1}{WH}\sum_{k=1}^{WH}}}\begin{array}{c} \max\\ \underset{CCMP}{\underset{︸}{j=1,2,\ldots ,\xi }} \end{array}\underset{CWA}{\underset{︸}{\left[M_{i}\cdot F_{i,j,k}\right]}}$$

The discriminative component is used to force feature channels to align with class information, and each feature channel corresponding to a particular class should be sufficiently discriminative, which includes four important components. Channel-Wise Attention (CWA), which denotes channel attention, is the process of taking the channel corresponding to each class and discarding it randomly; Cross-Channel Max Pooling (CCMP), which pools all discriminable features of each class into a one-dimensional feature map. Global Average Pooling (GAP), global average pooling, calculates the average response of each feature channel to obtain a C-dimensional vector where each element corresponds to a separate class. Finally, Softmax, for classification.

Diversity component *L*_*div*_ is defined as follows:


(7) $$L_{div}(F)\;=\;\frac{1}{c}\sum_{i=0}^{c-1}h(F_{i})$$

(8) $$h(F_{i})=\sum_{k=1}^{WH}\begin{array}{c} \max\\ \underset{CCMP}{\underset{︸}{j=1,2,\ldots ,\xi }} \end{array}\underset{CWA}{\underset{︸}{\left[\frac{e^{F_{i,j.k}}}{\sum_{k^{\prime }=1}^{WH}\;e^{F_{i,j.k^{\prime }}}}\right]}}$$

Polynomial components are used in order to make the variability between each component of the feature map F greater and to obtain as many diverse features as possible. It consists of four main components: Softmax, which acts as a spatial dimension normalization; CCMP, which is a cross-channel maximum pooling; Sum, which sums all elements on each feature map; and Average, which averages the values of all channels.

### EMRES-50

In the clothing images shown on major shopping sites, the collar region accounts for a small proportion of the whole image, and the arbitrary angle of the shot usually makes the collar region appear distorted, missing, and other features. The use of classical convolutional neural networks to process this kind of image data cannot effectively allow deep learning models to focus more on a piece of certain local information. The attention mechanism network can be used to emphasize or select the important information of the target processing object and suppress some irrelevant detailed information. ResNet (He et al., 2016) effectively solves the degradation problem triggered by increasing depth in deep neural networks due to easy optimization and residual blocks using jump connections, making it easy for the network to learn constant mappings and keep performance without degradation. Therefore, the ResNet network has become a mainstream model in the image field. ECA-ResNet50 is an improvement on ResNet by applying the attention mechanism ECA module to the residual block, which effectively channels the attention learning mechanism makes the network's image feature extraction capability improved. The traditional cross-entropy loss function is the most commonly used loss function in classification, which is used to measure the difference between the distribution learned by the model and the true distribution. Although the cross-entropy function uses an inter-class competition mechanism, which only cares about the accuracy of the prediction probability for the correct label and is good at learning information between classes, it ignores the differences of other non-correct labels, resulting in the learned features being more scattered and only focusing on the global information, which cannot classify and recognize the smaller collar regions in the collar image well. The main idea of fine-grained classification is to identify distinguishable features among subclasses. MC-Loss drills down on the channels to effectively navigate the model, focusing on different distinguishing regions and highlighting diverse features. At the same time, Mutual-Channel Loss does not require any fine-grained qualifying boxes or component annotations and can be combined with cross-entropy loss on commonly used network structures to enhance the network classification ability during the network training phase.

Based on the combination of ECA-ResNet50 and MC-Loss, this paper proposes a fine-grained image classification model—EMRes-50 based on a small collar dataset. EMRes-50 model, due to the addition of MC-Loss, enables the model to continuously focus and distinguish distinguishable regions and discriminable regions from the channel aspect, which can effectively avoid the problem that the features learned by the traditional cross-entropy function are not strongly distinguishable when dealing with fine-grained image classification. So, it can effectively deal with the fine granularity classification of collar images in complex backgrounds. The architecture of the main feature extraction modules of EMRes-50 is shown in Table 1. After each residual block in the original ResNet, the ECA module is added to aggregate multi-scale contextual information from the channels.

**Table 1:** EMRes-50 network structure.

	EMRes-50 network structure	
	7 × 7 conv 64	
	3 × 3 conv 64	
C:1 × 1	Conv 64	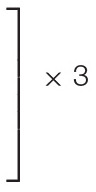
C:3 × 3	Conv 64	
C:1 × 1	Conv 256	
ECA module	256	
C:1 × 1	Conv 128	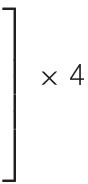
C:3 × 3	Conv 128	
C:1 × 1	Conv 256	
ECA module	256	
C:1 × 1	Conv 256	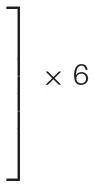
C:3 × 3	Conv 256	
C:1 × 1	Conv 1,024	
ECA module	1,024	
C:1 × 1	Conv 512	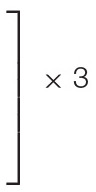
C:3 × 3	Conv 512	
C:1 × 1	Conv 2,048	
ECA module	2,048	
C:1 × 1	Conv 2,200

*Average pool, 6d, fc, softmax*.

**Figure 4:**
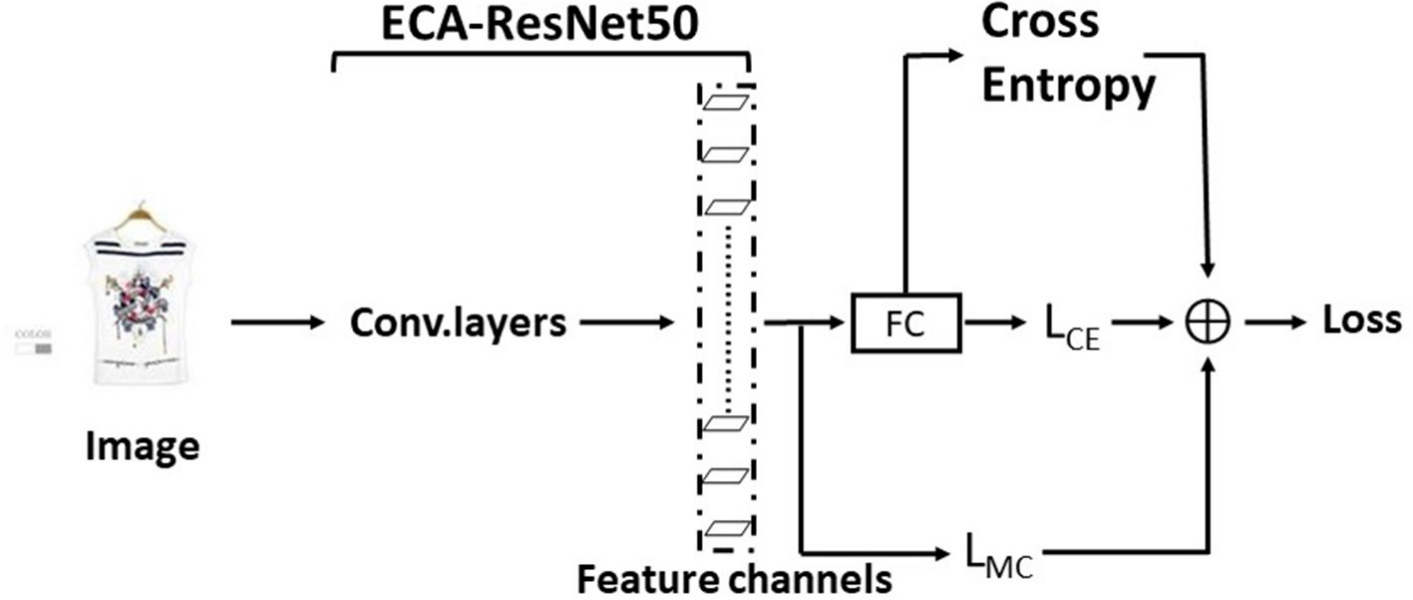
Schematic diagram of EMRes-50 training phase architecture.

The overall network architecture of the training phase of EMRes-50 is shown in Figure 4. The interoperability channel loss takes the output feature channels as input and uses hyperparametric support with the cross-entropy loss function assembled together to guide the update of the weights during the training phase, making the model output more diverse features for each class and a more pronounced feature gap between classes.

The weight update process is shown in Table 2. The weight update of EMRes-50 is divided into two steps. Through these two steps, the weight update of the entire network is completed:

**Table 2:** EMRes-50 weight update process.

**Algorithm: EMRes-50 weighting update process**
Input: Let the network have N layers, *W*_*n*_ is the weight parameter of the last FC layer, *W*_*i*_ is the weight parameter of a layer of the network,*i*∈1……*n*,
1: *While*(*i* < = *n*)
2: *if i* = 1……*n*−1
3: $W_{i}=W_{i}-\lambda \frac{\vartheta \left(L_{CE}+\mu L_{MC}\right)}{\vartheta W_{i}}$ //Except for FC, other weight layers are combined by cross-entropy loss and MC-Loss
4: *if i* = *n*
5: $W_{n}=W_{n}-\lambda \frac{\vartheta L_{CE}}{\vartheta W_{n}}$ //The last FC layer uses the traditional cross-entropy loss to update the weights
6: Updata by*W*_*i*_
7: Updata by*W*_*n*_
8: End

The first step: In addition to the fully connected layer, other weight layers are combined with the cross-entropy loss and MC-Loss to obtain the weight *W*_*i*_, and update *W*_*i*_ through the loop network layer *N*.

Step 2: The last fully connected layer uses the traditional cross-entropy loss to update the weight *W*_*n*_, and updates *W*_*n*_ through the loop network layer *N*.

## Experiment

### Dataset

This paper constructs a clothing collar type dataset named Collar-6, the images are from Taobao (https://www.taobao.com/), Tmall (https://www.tmall.com/), clothing brand official websites, and other major e-commerce platforms, through the manual collection, crawler way to collect the images collected by the figure. The images are used for experimental purposes only, not for commercial use.

**Figure 5:**
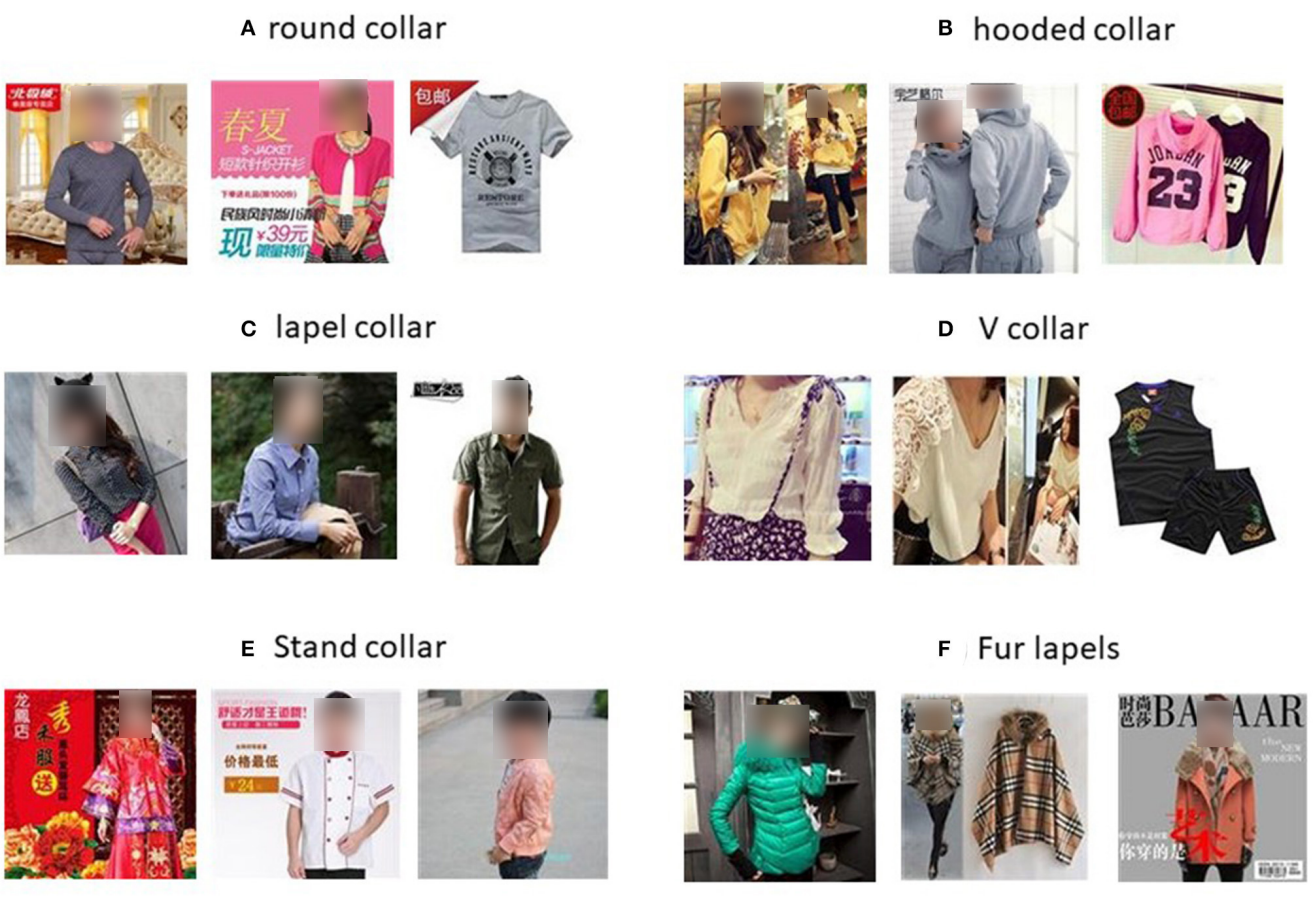
Some images from the Collar-6 dataset.

The Collar-6 dataset contains 6 categories: round collar, lapel collar, stand collar, hooded collar, V collar, and fur lapel, with men's, women's, and children's clothing, with a total of 18,847 images. The collar part in most of the images only occupies a small part of the image, and the rest of the area belongs to the noise which is not related to classification. Therefore, this dataset is difficult to classify images with rich diversity, which helps to learn the features of collars. Table 3 shows the distribution of the number of images per category in the training and test sets and the total number of that category, each containing about 3,000 RGB of three-channel images. Figure 5 shows some images of the six categories of collar types.

**Table 3:** Collar-6 experimental data distribution.

**Collar type**	**Number of training set images**	**Number of test set images**	**Total**
Round collar	2,480	620	3,100
Lapel collar	2,608	652	3,260
Stand collar	2,464	616	3,080
Hooded collar	2,560	640	3,200
V collar	2,468	617	3,085
Fur lapels	3,122	625	3,122

In order to verify the effectiveness of the classification algorithm proposed in this paper, DeepFashion (Liu et al., 2016), a large publicly available apparel image dataset, is used to verify the effectiveness of the classification algorithm. DeepFashion (http://mmlab.ie.cuhk.edu.hk/projects/DeepFashion.html) is a large-scale apparel dataset open to the Chinese University of Hong Kong, with 800,000 images, which contain images from different angles, different scenes, buyer shows, buyer shows, etc. Due to a large number of images in the dataset, and the amount of data in some categories is not large, in order to ensure that the size of the collected images is equivalent to the size of the Collar-6 dataset, the DeepFashion dataset is extracted by the following folder keywords: Dress, Jacket, Jeans, Shorts, Tank, Tee 6 categories, a total of 18,727 images for experimentation. The distribution of experimental data of DeepFashion-6 is shown in Table 4.

**Table 4:** Distribution of experimental data of DeepFashion-6.

**Type**	**Number of training set images**	**Number of test set images**	**Total**
Dress	2,555	639	3,194
Jacket	2,505	627	3,132
Jeans	2,412	603	3,015
Shorts	2,541	636	3,177
Tank	2,528	632	3,160
Tee	2,439	610	3,049

### Experimental Setup

All model experiments in this paper are trained on Intel i7-7700 processor, 1T SSD, 64 RAM, and NVIDIA GTX2080Ti GPU, using the pytorch framework. Stochastic gradient descent (SGD) is used as the optimization method. The number of iterations is 300. The initial learning rate is set to 1e-2, and the learning rate is adjusted to 1e-3 when the iteration reaches 150. the batch size is set to 32. the size of all images for the experiments is uniformly 224 × 224. the comparison networks are compared with EMRes-50 using the cross-entropy loss function in the comparison experiments.

### Analysis of Experimental Results

#### Collar Image Classification Experiments Based on the Collar-6 Dataset

In order to solve the problems of unsatisfactory classification of collar images and imprecise collar feature extraction by traditional convolutional neural networks, the EMRes-50 method is proposed, which adds MC-Loss to the existing channel attention module of the ECA-ResNet network to further ensure that the network focuses as much as possible on the discriminative part and the discriminative part, thus helping the network to perform fine-grained feature learning.

A comparison of the classification accuracy of EMRes-50 with a variant of ResNet or a ResNet-based improved model on Collar-6 is shown in Table 5.

**Table 5:** Comparison with variants of ResNet or improved models based on ResNet.

**Models**	**Accuracy %**
ResNeXt50	73.05
CBAEMRes-50	63.68
SE-ResNet50	63.44
SCNet50	66.07
Res2Net50	73.44
**EMRes-50**	**73.60**

As can be seen from Table 5, the classification accuracy of EMRes-50 on the Collar-6 dataset obtained the highest in comparison with a variant of ResNet or a model based on ResNet improvements.ResNeXt50 (Xie et al., 2017), SCNet50 (Liu et al., 2020), and Res2Net50 (Gao et al., 2019) are all variants of the ResNet network.ResNeXt50 utilizes group convolution, constructing a parallel stack of blocks with the same topology, and is a simple, highly modular network structure for image classification, but its composition is limited by stacking blocks with the same specifications, and is able to extract The classification accuracy is 73.05%, which is 0.55% lower than that of EMRes-50. Res2Net is designed with finer-grained layer blocks in order to extract more multi-scale features, increasing the range of perceptual fields in each layer, and has a strong multi-scale representation capability, which is suitable for extracting collar images of different scales. However, it ignores the relationship between the global and local position of the collar part in the whole image, so the classification accuracy of Res2Net50 is 73.44%. EMRes-50 introduces MC-Loss based on ECA-ResNet50, which, together with cross-entropy, can make the network focus on both global discriminative regions and local discriminative regions, and the classification accuracy is 0.16% higher than that of Res2Net50 is 0.16% higher than Res2Net50. SCNet50 can effectively improve the range of sensory field through self-correction operation and help the network generate more discriminative feature expressions, but it only takes into account the inter-channel information and local information without considering the global location information, and the effect of classification for collar images is rather poor, with an accuracy of only 66.07%.CBAM (Woo et al., 2018) and SE (Hu et al., 2018) are two classical attention mechanism models, but introducing the attention mechanism only on ResNet50, although it can focus on the collar part, it cannot distinguish the low-level distinguishable features such as different collar edges well, and the classification effect is not satisfactory, only 63.68 and 63.44% accuracy respectively, which are 9.92 and 10.16%.

A comparison of the classification accuracy of EMRes-50 and the lightweight convolutional neural network model on Collar-6 is shown in Table 6.

**Table 6:** Comparison with lightweight model.

**Models**	**Accuracy %**
MobileNetV3_large	68.48
MobileNetV3_small	63.23
GhostNet	60.98
SqueezeNet1_0	63.76
**EMRes-50**	**73.60**

As can be seen from Table 6, on the Collar-6 dataset, EMRes-50 obtains better classification accuracy on collar images in comparison with the lightweight model. GhostNet (Han et al., 2020) only considers from the perspective of generating feature maps, reducing the total number of parameters it needs and computational complexity, without enhancing the network extraction effect in terms of feature extraction capability, so the classification effect on collar images is The accuracy is only 60.98%, which is 12.62% lower than the accuracy of EMRes-50.The design of the Fire module in SqueezeNet (Iandola et al., 2016) performs model compression by reducing the parameters, and the mixture of 3 × 3 and 1 × 1 convolution increases the feature extraction capability of the whole model. However, the SqueezeNet network is not deep, and there are limitations in feature extraction for different types of collars, and the accuracy is 9.84% lower than EMRes-50, which yields 63.76% accuracy. MobileNetV3 (Howard et al., 2019), which uses a large number of 5 × 5 size convolutional kernels, is not good for fine-grained collar images because there are no multi-scale convolutional kernels used alternatively classification, MobileNetV3_large and MobileNetV3_small have 68.48 and 63.23% classification accuracy respectively, which are 5.12% and 10.37% less accurate than EMRes-50, respectively.

A comparison of the classification accuracy of EMRes-50 with other classical convolutional neural network models on Collar-6 is shown in Table 7.

**Table 7:** Comparison with other models.

**Models**	**Accuracy %**
AlexNet	67.53
Xception	70.53
VGG16	63.68
VGG19	65.62
**EMRes-50**	**73.60**

As can be seen from Table 7, EMRes-50 obtains better collar image classification accuracy in comparison with other classical convolutional neural network models on the Collar-6 dataset. AlexNet (Krizhevsky et al., 2012) and VGGNet (Simonyan and Zisserman, 2014) filters are both linear topologies, which means that these networks can only have relatively inflexible perceptual fields and obtain lower classification accuracies for both low, with 63.68% and 65.62% accuracy obtained by VGG16 and VGG19, respectively, and 67.53% accuracy obtained by AlexNet, both of which are lower than the accuracy obtained by EMRes-50.Conventional convolution is a direct extraction of spatial and channel information through a convolutional kernel. Xception (Chollet, 2017), on the other hand, is a convolutional neural network architecture based entirely on depth-separable convolutional layers. As an improved version of InceptionV3, it retains the network's multiscale feature extraction capability, and its model performance on collar image classification is better than AlexNet and VGGNet, obtaining an accuracy of 70.53%, but still 3.07% lower than the accuracy obtained by EMRes-50.

#### Validation Experiments

To verify the effectiveness of the EMRes-50 method class, the validity of the classification algorithm was verified using the publicly available large apparel image dataset DeepFashion. The experimental results are shown in Table 8.

**Table 8:** Comparison of model accuracy in the DeepFashion-6 dataset.

**Models**	**Accuracy %**
AlexNet	83.50
ResNet50_CBAM	82.78
GhostNet	82.84
InceptionV3	73.36
MobileNet_large	83.77
MobileNet_small	83.40
Res2Net	85.13
SCNet	79.57
SqueezeNet1_0	82.03
Xception	85.01
**EMRes-50**	**86.09**

It can be seen from Table 8 that the performance of EMRes-50 on DeepFashion-6 has a certain improvement compared with other convolutional neural networks because after the introduction of MC-Loss in the basic network, the network can capture the discriminative and identifiable performance. There are more distinguishing features, which improve the classification performance of the network to a certain extent. EMRes-50 is 0.08% higher than Xception, which has better accuracy and is higher than other convolutional neural networks. The results show that although EMRes-50 is designed primarily for the collar-6 Collar dataset, it can effectively categorize garment areas without collars while accurately identifying Collar areas. It shows that EMRes-50 can continuously find the distinguishable features of classified objects with different region proportions in an image through algorithm iteration, so as to improve the classification effect. At the same time, the DeepFashion-6 dataset is the same as the Collar-6 dataset. When the classified objects have different angles, different scenes, and other noises, the classification performance of EMRes-50 can still be compared to these two datasets. The improvement indicates that EMRes-50 has a better ability to distinguish the distinguished features. Such results fully verify that EMRes-50 not only has good classification performance on collar images but also shows good classification effects in the field of clothing image classification.

#### Ablation Experiments

##### Structural Ablation

The ablation experiments were conducted on two datasets, Collar-6 and DeepFashion-6, with Resnet50 as the base network, and the effects of introducing the attention mechanism ECA block and MC-Loss loss analysis on the experimental effects, respectively. Ablation experiments of EMRes-50 on the Collar-6 dataset are shown in Table 9. As can be seen from the table, EMRes-50 improves 14.24% compared to ResNet50, 6.76% compared to ResNet50 by introducing only MC-Loss, and 16.1% compared to ResNet50 by introducing only ECA block. Since the collar part is not solely present in the collar image, the non-collar part also occupies most of the space in the image, which can interfere with the training of the convolutional neural network. The accuracy of ResNet50 is 1.86% lower than that of ResNet50 when only the ECA module is introduced, indicating that the attention module makes the model focus too much on the overall part of the collar and cannot effectively guide the network to focus on the distinguishable areas of the collar, ignoring the differences between different collar types, while the introduction of the MCLoss loss function can consider more distinguishable areas of the collar and guide the network to perform the correct weight optimization for fine-grained classification. The introduction of the MCLoss loss function can guide the network to optimize the correct weights and greatly promote the network to learn the distinguishable local features for fine-grained classification, effectively avoiding the negative impact of the ECA attention module.

**Table 9:** Models for ablation experiments on the Collar-6 dataset.

**Models**	**Accuracy %**
ResNet50	59.36
ResNet50+MC-Loss	66.84
ResNet50+ECA	57.50
**EMRes-50**	**73.60**

EMRes-50 introduces both the ECA module and MCLoss, which makes the network not only focus on the collar region, but also highlight the local regions of different types of collars, and the negative effect brought by the ECA module to the network is transformed into a facilitating effect. Thus, the combined effect of both the ECA module and MC-loss further enhances the feature extraction capability of the network, resulting in a large improvement of the network performance.

The ablation experiments of EMRes-50 on the DeepFashion-6 dataset are shown in Table 10. As can be seen from the table, EMRes-50, compared to ResNet50 only introduced MC-Loss improved by 1.56%, and compared to ResNet50 only introduced ECA block improved by 5.15%. The non-category related part of the category image of clothing occupies a larger space of the image, which is not as disturbing to the training of the convolutional neural network as the collar image classification, but EMRes-50 can still bring an improvement in accuracy in the field of clothing image classification, verifying that EMRes-50 can effectively improve the network performance.

**Table 10:** Ablation experiments of the model on the DeepFashion-6 dataset.

**Models**	**Accuracy %**
ResNet50	81.47
ResNet50+MC-Loss	84.53
ResNet50+ECA	80.94
**EMRes-50**	**86.09**

##### Hyperparametric Ablation

Verify the effect of one-dimensional convolutional size *k* on EMRes-50 and the validity of *k* size selection. After channel-level global averaging pooling without dimensionality reduction, the ECA module captures local cross-channel interaction information by considering each channel and its *k* neighbors, where the convolutional kernel size of k represents the coverage of local cross-channel interactions, i.e., how many neighbors near that channel are involved in the attention prediction of this channel. In EMRes-50, *k* is set to 3, 5, and 7. The results are shown in Table 11. *k* = 3 gives the best results for EMRes-50, and the accuracy rate decreases with larger *k*, indicating that for the collar region, which accounts for a smaller percentage of the collar image, the smaller the value of 1D convolution, the easier it is to capture the features of the region and improve the accuracy rate. On the contrary, the larger the value of 1D convolution is, the more noise is introduced, which affects the recognition of collar regions by the network and makes the accuracy rate decrease.

**Table 11:** Different effects of different k on the ECA module on the Collar-6 dataset.

**k**	**Accuracy %**
5	70.82
7	67.37
**3**	**73.60**

## Conclusion

In the context of the development of apparel e-commerce, efficient and accurate collar classification is beneficial to merchants for apparel information description, convenient for a wide range of consumers to shop using keyword queries, and promotes the development of the apparel sales industry. In the absence of related research, this paper constructs a collar dataset named Collar-6 and the images contain a lot of noise. Based on ECA-ResNet50 and the introduction of MC-Loss, this paper proposes a fine-grained image classification model based on a small collar dataset, called EMRes-50. Comparative experiments and ablation experiments are conducted on the Collar-6 dataset, and the results show that EMRes-50 can effectively improve the classification performance of the underlying network ECA-ResNet50, and outperforms most classical and novel classification models in recent years, indicating that EMRes-50 can effectively solve the fine-grained collar image classification problem, and the extracted features are more differentiable and enhance the model classification effect. On the other hand, to verify the effectiveness of EMRes-50, comparison experiments are conducted on the public dataset DeepFashion, and EMRes-50 is still able to improve the classification effect of garment image classification, indicating that EMRes-50 can be applied not only to the field of collar image classification but also extended to the field of garment image classification.

## Author's Note

The types of clothing are increasing day by day, and it is becoming more and more important to classify clothing according to its collar design. Nowadays, popular shopping websites all support keyword search for the clothing styles you want to buy, including clothing collar keyword search. However, the product information of the website is often described in the form of a combination of direct image description and key text annotations. If it can be directly described through images, a lot of time and labor costs can be reduced. In addition, the collar part occupies a small proportion in the entire image. Such image classification is more challenging than ordinary image classification. At present, there are few researches on collar classification and related data sets. Therefore, this paper constructs a six-category small collected data set, and builds a model named EMRes-50 for this data set, and proves the improvement of the model through experiments. It can effectively solve the problem of collar image classification.

## Data Availability Statement

The raw data supporting the conclusions of this article will be made available by the authors, without undue reservation.

## Author Contributions

All authors listed have made a substantial, direct, and intellectual contribution to the work and approved it for publication.

## Funding

This work was partially supported by the National Natural Science Foundation of China under Grant Nos. 61962006, 61802035, and 61772091; the Project of Science Research and Technology Development in Guangxi under Grant Nos. AA18118047, AD18126015, and AB16380272; thanks to the support by the BAGUI Scholar Program of Guangxi Zhuang Autonomous Region of China [2016(21), 2019(79)]; the National Natural Science Foundation of Guangxi under Grant Nos. 2018GXNSFAA138005; the Sichuan Science and Technology Program under Grant Nos. 2018JY0448, 2019YFG0106, and 2019YFS0067; Guangxi University Young and Middle-aged Teachers Scientific Research Basic Ability Improvement Project (Grant Nos. 2020KY04031. Project Name: Research on Key Technologies of Intelligent Data Processing in Active Distribution Network environment).

## Conflict of Interest

The authors declare that the research was conducted in the absence of any commercial or financial relationships that could be construed as a potential conflict of interest.

## Publisher's Note

All claims expressed in this article are solely those of the authors and do not necessarily represent those of their affiliated organizations, or those of the publisher, the editors and the reviewers. Any product that may be evaluated in this article, or claim that may be made by its manufacturer, is not guaranteed or endorsed by the publisher.
